# Reducing locoregional treatments in low risk early invasive breast cancer

**DOI:** 10.1016/j.ejso.2026.111411

**Published:** 2026-01-16

**Authors:** Shelley Potter, Mairead MacKenzie, Stuart McIntosh, Toral Gathani, Sileida Oliveros, David Dodwell

**Affiliations:** a University of Bristol, UK; b Independent Cancer Patients’ Voice, UK; c University of Belfast, UK; d University of Oxford, UK

## Abstract

Improved outcomes, particularly the marked reduction in locoregional recurrence, and the increasing numbers of survivors of early breast cancer, have led to the wish to reduce treatment morbidity, without compromising long-term outcomes.

Trials to assess reduced breast and axillary surgery, radiotherapy and endocrine therapy have all been conducted and led to new standards of care and reduced toxicity.

In some cases, however, implementation of research findings has been compromised by a relative lack of quality control in trials, substitution of a de-escalated treatment by an alternative, a lack of patient-relevant endpoints and the difficulties in the design and powering of ‘non-inferiority’ trials.

We summarize the current landscape of care and clinical research in this area, explore the problems identified in some studies and suggest how they may be mitigated.

## 1 Introduction

The clear reductions in mortality and local recurrence for patients with early invasive breast cancer (EIBC) in high resource countries, seen over the last three decades is a resounding public health success story [1]. These improvements are the result of decades of painstaking clinical research.

Increasing numbers of women now present with low stage disease, driven by effective strategies for early detection and diagnosis. The majority are treated by breast conserving therapy (BCT – breast conserving surgery (BCS) and breast radiotherapy (RT)) with sentinel lymph node biopsy (SLNB) for axillary staging. Most have oestrogen receptor (ER) positive disease and also receive a recommendation for ≥5 years of endocrine therapy.

The widespread use of BCT is associated with low risks of local recurrence, and improved quality of life (QoL) for rapidly increasing numbers of patients, but treatment morbidity remains.

Chest wall, breast and rib pain are common after mastectomy and BCT; cosmetic outcomes after BCT can be poor and axillary lymph node dissection (ALND), currently recommended for patients with node positive disease, causes intractable lymphoedema in 20–30 % of women and chronic arm pain in 2–5 %. SLNB has smaller but definite risks of these serious morbidities. Endocrine therapy also has underappreciated toxicities, related to oestrogen deprivation [2].

Efforts to reduce long-term toxicities are important, but so is avoidance of the distressing symptoms of locally recurrent disease which was so prevalent in past decades and is now uncommon.

As such, despite much improved long-term cancer outcomes, this ‘one size fits all’ approach to locoregional therapy (LRT) has come under question because of concerns around the morbidity and costs of overtreatment.

Clinical trials, designed to optimise locoregional management, have explored omission or reduction in the extent of breast and axillary surgery, RT and endocrine therapy.

We discuss current care options and research findings in this area and some issues that have arisen in the design, conduct and interpretation of recent clinical trials.

## 2 Treatment standardisation

The design of trials of treatment reduction should focus on the reduction of one treatment and discourage the addition or subtraction of non-trial therapies, as trial results will become difficult to interpret.

Recent randomised trials of reduced axillary surgery have been affected by concerns about compensatory addition of RT in some circumstances and that results may relate to a substitution, rather than reduction, of surgery [3–6]. This has created uncertainty in the introduction of this evidence into care. Quality assurance for both axillary surgery and RT are necessary in future clinical trials and in routine care.

Conversely in patients randomised to RT vs reduced or no RT after BCS, there may be an inclination to omit endocrine therapy, with such omission being more likely in patients who received standard RT, resulting in a risk of confounding and uncertainty in the application of trial results.

## 3 Adherence to endocrine therapy

In ER-positive breast cancer endocrine therapy reduces both breast cancer mortality, distant metastases, and locoregional recurrence. Following BCS, UK NICE guidelines recommend consideration of partial breast RT or RT omission in some categories of patients at very low risk of local recurrence, but only in patients who will receive 5 years of endocrine therapy. However, adherence to endocrine therapy cannot be predicted at treatment initiation and has been reported to be around 50–70 % at 5 years [2]. Trials of de-escalated LRT are similarly dependent on an expectation of compliance with a systemic treatment which around 30–40 % of patients may be unable to tolerate.

An interim analysis of the EUROPA trial which randomised women >70 years to breast RT or endocrine therapy after BCS, demonstrated significantly fewer adverse events and improved QoL in those who received RT [7]. This finding again reflects the toxicity of, and concerns about adherence to, endocrine therapy. It is thus reasonable to consider reduced systemic rather than locoregional therapy within the overall ambition of preventing treatment toxicity.

## 4 Trial end-points

Reductions in morbidity within randomized clinical trials of reduced locoregional treatment may be realised within a few years, but the long natural history of low-risk early breast cancer and risk of late relapse, is problematic because of low event rates and the need for unfeasibly large sample sizes to derive oncological outcomes.

There are no easy solutions but improved international collaboration, with a view to future patient-level meta-analysis, harmonisation of trial protocols, consistent end-point definitions, and prioritisation, by patients, of toxicity outcomes, are needed. Setting a trial within a well-defined prospective cohort with similar demographic and disease-related characteristics and similar access to care, to enable the collection of more data on cancer outcomes, beyond the trial population, is an option.

## 5 Patient and public involvement

Trials that set out to test treatment reduction are an exploration of the trade-off between an anticipated lessening of morbidity against a higher risk of local recurrence. Careful trial design and oversight are needed but cannot eliminate risk. Enthusiastic patient partnership, from the earliest stages of trial development and subsequent conduct, to inform these trade-offs and to select meaningful endpoints, is paramount.

The James Lind Alliance supports patient involvement in clinical research and has described a number of priorities for surgical treatment in EIBC. The highest ranked of these was a need to explore avoidance of ALND [8].

## 6 The current landscape of care and research

Currently the approach of BCT in low risk EIBC, without nodal involvement has moved to include the use of partial breast RT or RT omission in some circumstances, but initial surgical excision, SLNB, and five years of endocrine therapy in ER +disease remains standard.

Ongoing clinical research may in future support the use of non-operative image-guided excision [9] or ablative techniques eg cryotherapy or high-intensity focussed ultrasound (HIFU) [10] under local anaesthesia to remove or ablate small breast tumours.

The routine use of SLNB in patients with radiologically node negative disease is also under question. The SOUND [11] and INSEMA [12] trials provide indicative evidence that patients with radiologically node negative cancers ≤ 20 mm in size may not derive benefit from SLNB. It is likely that the avoidance of any form of axillary surgery will, for many patients, soon become standard.

In pathologically node positive disease, identified following SLNB, ALND, axillary radiotherapy or avoidance of further axillary treatment, are variably recommended. The latter two options avoid the high risk of arm lymphoedema but sacrifice some information on nodal stage which can be clinically informative.

Patients with axillary node positive disease at diagnosis are usually recommended to have ALND. Of all locoregional treatments this is arguably associated with the greatest risk of morbidity, and an exploration of whether it is always needed is ongoing. Targeted axillary dissection (TAD) in which the lymph nodes containing cancer, and the first draining (sentinel) lymph nodes are removed is a promising technique which may reduce lymphoedema risk, but randomized evidence is needed to change care [13].

## Conclusion

The morbidity and cost of overtreatment is a recognised problem in the contemporary management of EIBC. Research to explore reductions in the use and extent of surgery, RT and systemic therapy for the increasing number of women with low risk disease, presents major challenges but patients and clinicians are agreed is a priority.
